# Chemical 24/7

**DOI:** 10.1007/978-3-030-57081-1_6

**Published:** 2020-10-14

**Authors:** Anita Hardon

**Affiliations:** University of Amsterdam, Amsterdam, Noord-Holland the Netherlands

## Abstract

This chapter shines a light on what happens in the dark: specifically, we present ethnographic insights from the nightlife economy and how chemicals enable youth to work “24/7.” Producers, promoters, DJs, hosts, artists, performers, drag queens, musicians, stage managers, bartenders, hospitality girls, and dancers from Amsterdam, Brooklyn, Bira (Indonesia), and Puerto Princesa (the Philippines) share with the ChemicalYouth team the various stimulants they use to stay awake and perform their jobs during non-typical working hours, and the other chemicals that they take in order to be able to sleep and recover afterwards. In *Chemical 24/7* we compare and contrast the chemical practices of youth working at leisure industry sites in the global North to those of the low-income service sector and manual workers in the global South, and discuss how these different working conditions perpetuate chemical use. Our interlocutors rely on a range of chemicals for their work and social lives, and they develop practices to moderate their use in order to avoid adverse effects. Yet their practices differ depending on the availability, marketing, and policing of the substances.

We have so many ethnographic stories to tell about how young people use chemicals for energy, so many, that I have had a hard time developing a structure for this chapter. Having just moved back from to the Netherlands from the United States because of the coronavirus pandemic, I’m also still a bit jetlagged. I need a coffee. Thinking this, I’m reminded of a conversation I had with one of the youth ethnographers, Daan Kamps, a student and DJ in the Amsterdam nightlife scene, which he studied for the ChemicalYouth project. A few years ago, we were having lunch to discuss his interviews and observations, and I had just returned from a fieldwork visit to the Philippines. “I need a coffee,” I told him. I’m jetlagged. Having just completed a course on chronobiology to educate himself on the circadian rhythms of humans, he lectured me on the health risks of jetlag: it can impair cognition, concentration, and motor coordination as well as cause metabolic disorders, such as obesity and high blood pressure (Roenneberg 2012). I should walk outside in the sun, he tells me. Real sunlight helps.

Nightwork—meaning all jobs that are executed during nighttime—increased as cities became connected to electricity in the early twentieth century, and as economies became more connected globally through the internet. In the current “ gig economy”—dominated by those jobs in which people are paid by the task, rather than receiving a fixed salary— work never stops. Part of this “ performance” has to do with adapting to the different temporal demands of the labor market—in other words, the management of sleep and wakefulness. As Crary (2013, p. 17) notes, our modern economies “[undermine] distinction between day and night, between light and dark, and between action and repose … the planet becomes re-imagined as a non-stop work site or an always open shopping mall of infinite choices, tasks, selections, and digression.”

But working at night is not necessarily good for us. Melatonin, the hormone involved in the regulation of our biological clock, is released when we are exposed to daylight. Our bodies follow circadian rhythms of approximately 24-hour cycles, and as Kamps warned me, these rhythms have an impact on our mental and metabolic health (Roenneberg et al. 2003).

Humans have a long history using chemicals to tinker with our circadian rhythms, with caffeine being the most ubiquitous substance used to stay awake, one that is accepted globally as beneficial despite its addictive properties. Historians trace the use of coffee back to the fifth century, in the Sufi monasteries of Mocha, now known as Yemen (Weinberg and Bealer 2001). Caffeine stimulates the central nervous system by blocking the action of adenosine (which causes drowsiness) on its receptors. The popular food writer Pollan (2020), in a recent analysis of caffeine, suggests that without this substance the industrial revolution wouldn’t have happened. Studies in sports medicine attest to caffeine’s positive effects in adults, including increased endurance and strength, improved reaction time, and delayed fatigue (Graham 2001; Sökmen et al. 2008). Adverse effects of caffeine, if taken in high amounts, include disturbed sleep, increased blood pressure, and physical addiction. Caffeine is considered safe to ingest up to about 150 mg per day (or two cups of coffee).

In addition to caffeine, those working at night often resort to cocaine and amphetamines for stamina and to stay awake. The use of cocaine for endurance goes back to the Incas in Peru who for thousands of years have chewed coca leaves for this purpose. The alkaloid cocaine, derived from the plant, was only isolated in the mid-nineteenth century. Amphetamines were discovered shortly thereafter by chemists. In *On Speed* Rasmussen (2008) traces the history of this category of drugs, showing how early twentieth-century pharmacies in the United States sold invigorating tonics containing cocaine and nasal decongestants containing amphetamines. Its first major use was during World War II, when soldiers used it to boost their performance and alertness, and to suppress appetite (see also Braswell 2005; Rawson et al. 2006). In Japan, it was given to soldiers before they performed their “kamikaze” suicide bombing missions; in England, 73 million amphetamine tablets were made available to pilots so they would not fall asleep (Braswell 2005). Three years after the war, in 1948, the Japanese Ministry of Health prohibited the production of both tablet and powder form of methamphetamine. Similar moves were made in the United States but, interestingly, the medical establishment continued to defend its legal status and deny its addictive potential. It was only when its use grew further, and more evidence about its addictive potential came to light, that the United States finally passed the 1974 Drug Control Act (Rawson et al. 2006).

Nowadays cocaine is an illegal drug. Amphetamines are sold legally as prescription tablets for ADHD, while methamphetamine is an illegal drug, sold on the street as “crystal” and “ice,” and consumed in many different ways: ingested pills, snorted powder, injected liquid, or smoked (Rawson et al. 2006). Like caffeine, cocaine and methamphetamines increase alertness, strength, and coordination, while also increasing blood pressure and stimulating the heart rate (Koob et al. 2004). Both drugs cause pupils to dilate, allowing more light in, which activates our daytime metabolism (Paul et al. 2009). The pharmacological actions of cocaine and methamphetamine differ: cocaine works fast and is quickly removed from the body, while methamphetamines have a much longer duration of action, making it harder to sleep after using them and increasing the risk of addiction. Cocaine prolongs dopamine action in nerve cells, by blocking dopamine reabsorption, while methamphetamines work by both blocking dopamine re-uptake and increasing its release, leading to higher concentrations of the neurotransmitter in cells, which can be toxic. Long-term adverse effects of methamphetamines include insomnia, confusion, memory loss, and psychiatric conditions such as psychosis (National Institute on Drug Abuse 2019).

Social scientists have pointed to the widespread use of caffeine-containing “ energy drinks” and the off-label use of drugs indicated for attention deficit hyperactive disorder (ADHD), such as Ritalin and dexamphetamine, by students trying to increase their focus and attention when studying, and to stay up late when partying. But these studies generally focus on student populations. In this chapter, we present ethnographic insights on the use of various stimulants to stay awake and fight fatigue by workers in the nighttime leisure economy, the bartenders, DJs, sound technicians, photographers, and event organizers who must perform professionally during the night. We contrast the 24/7 chemical practices in Brooklyn and Amsterdam, two leisure industry sites in the global North, with the 24/7 chemical practices of low-income service sector and manual workers in our field sites in the global South: Puerto Princesa and Cagayan de Oro (the Philippines), and Makassar (Indonesia). Here the substances are not only used to stay awake, but also to endure heavy physical labor. We find that practices differ depending on the availability, marketing, and policing of the various legal and illegal stimulants, and that risks are shaped by the structure of everyday life, the rhythms of stimulant use, and workplace policies. We also show how—across the sites—users moderate intake in order to avoid adverse effects, including their negative influence on the quality of sleep, and how they mitigate such effects by using other substances, such as cannabis or alcohol, to calm down.

## Staying Awake in the Leisure Industry

In Brooklyn and Amsterdam our youth ethnographers conducted focused research on the nighttime leisure industry, with Tait Mandler studying DIY queer parties in Brooklyn and Daan Kamps studying popular clubs in Amsterdam. In these field sites, event managers organize the artists, barkeepers provide drinks, DJs create music, and sound technicians fine tune it. Mandler and Kamps found that the workers in these entertainment spaces balanced their engagement in the party and staying fit for work. All moderated their alcohol intake and they took stimulants to stay alert till the end of the night. Mandler (2016) observes that work in these sites is an inherently contradictory activity. When working, they have to appear to be enjoying themselves; leisure activities themselves become work. Cocaine helps workers be productive and enjoy themselves in their nighttime jobs.

Our interlocutors in these two cities presented themselves as experienced moderators of circadian rhythms. They had developed personal strategies of stimulant use, aimed at generating the right amount of alertness, while also enabling pleasure at work. They scheduled their use around work and other responsibilities, monitored their dosage to ensure moderation, and took breaks from using when necessary. But all attested to the heavy toll that nightwork has on their bodies, especially if they combine nightwork with daytime jobs, or coursework, both of which require rapid transitions from night wakefulness to daytime alertness.

In Brooklyn, Mandler (2016, 2018) found that the most common chemicals used to stay awake during the night shift were energy drinks, cocaine, and the ADHD drug Adderall. Cocaine helped his interlocutors stay alert during shifts, while also engaging in the party, which was a challenge. Jett, a bartender, said that after working in a bar for around six months, he realized that everyone around him was sniffing cocaine all the time. Jett preferred energy drinks because they were free to employees in the establishment (which was sponsored by an energy drink manufacturer). Mandler (2018) also found that many night workers combined day and nighttime jobs. Joshua, a drag queen and visual artist, described how they (Joshua’s preferred pronoun) managed working 20 hours in two days with only a few hours of sleep:

That morning I go to work all day, so I’ll have a coffee there. I get home around 6:30 pm or 7 pm. … [I] get to the club around 10:30 pm … and drink a big non-alcoholic drink to bookend the night with. Like seltzer and a fruit juice … Then right around midnight I start drinking … something like vodka with a fruit mixer. Throughout the night I’ll probably have two and half of those. Maybe someone had coke and I’ve done a bump [small snort] or two. Around 3 am or 3:30 am I’ll have left the club … I’ll sleep for three and half or four hours before I have to get up and go to work where I’ll drink a little coffee. (Mandler 2018, p. 263)

Alice produced underground queer events in warehouses that usually started around 11 pm and continued well past 4 am. She also worked for a nightlife magazine and was active on social media, which has made her a popular party host. Alice explained that she did not really enjoy the nightlife culture and was mainly interested in her own friends. However, her job was to promote events as if they were going to make for the best night ever. She didn’t drink much at these parties, but made sure to hold a drink; this made her feel as if she was engaged in the party, and dissuaded people from asking why she was not drinking. If she consumed alcohol, she said, she could not produce the parties well, and she would be tired before they were over. Cocaine, in contrast, helped her be more assertive, “but sometimes this also [led] her to stand on the stage at the end of a party and watch the crowd dance to the final songs in awe” (Mandler 2018, p. 2).

For Cassandra, and many other nightlife workers, the use of cocaine was not the event of the night. They moderated use, spacing and sharing it, as Dan explains:

I take smaller bumps, I try not to do a whole line. I space it out throughout the night. Also sharing kind of helps. If I’m going to do some and I have someone else to do it with, it sort of tricks me. (Mandler 2016, p. 269)

Val explained that he preferred cocaine because it:

doesn’t really affect my ability to judge … so that’s sort of the stimulant choice. I don’t like coffee; it wrecks my stomach. I hate things like Adderall, they’re too strong. Cocaine is light and it can keep me awake and after I get home I’ll fall asleep maybe an hour later. (Mandler 2016, p. 263)

Moderating their substance use was a common goal among these nightlife workers. They monitored the chemical use of their friends, specifically checking if it negatively affected their ability to function on the job or socially; and used the same criteria to evaluate their own chemical practice. Some limited themselves to a few drinks or bumps. Even so, at times, they felt that they had gone too far, at which point they took a break from using a specific drug, or even all chemicals (Mandler 2016).

Kamps interviewed 24 people who work in Amsterdam clubs. He recorded systematically which stimulants they used to stay alert and awake at work. Out of the 24 participants, 23 drank energy drinks and/or Coca Cola, 16 consumed amphetamines ( including ADHD drugs), and 15 used cocaine. As was the case in Brooklyn, the workers he spoke with asserted that they had developed personal strategies to stay awake and alert on the job. Many smoked cigarettes and drank alcohol; the two go well together, they said. Alcohol helped them feel more engaged with and connected to the clients at the clubs. But they felt compelled to moderate their alcohol consumption so as not to become too drunk to work.

Event organizers and venue programmers are responsible for the overall success of the party. They pick up the DJs, manage the guest list, decorate the venue, and are present for the sound check. They feel responsible for getting the dance floor filled and creating the euphoric ambience. Maarten, a venue manager used cocaine to stay alert and be sociable when preparing for events and to tune in with the atmosphere of the party. Meike, an event organizer and hostess, said she often worked long, 10-hour shifts, till 8 am the next morning. Besides taking smoking breaks, she often drank Club Mate, which she combined with vodka. Club Mate is an energy drink with 50 mg caffeine per 250 ml and low sugar content (less sugar than Red Bull). Nadine, too, who worked as a barkeeper, used cocaine for energy, alertness, and confidence.

DJs need to keep the crowd going through the night. Sander, a well-known DJ, talked about using ADHD drugs to keep up the vibe. His girlfriend had been diagnosed with ADHD and prescribed dexamphetamine. Before he left for work in the evening, Sander usually took two 20 mg tablets of dexamphetamine, and then took another dose if he felt tired during the night. Sander said that dextroamphetamine worked well: it kept him alert, without anyone noticing that he had taken drugs. The DJs interviewed by Kamps all attested to the highly desirable effects of amphetamines. The substances create very energetic states of being that contribute to the party atmosphere. The sound technician makes sure the music reaches the audience as well as possible. “Being alert and focused—paying continuous attention to sound levels, depth, clarity, and space—is of utmost importance, and the longer the event lasts, the greater the challenge,” explained one sound tech (Kamps and Hymans forthcoming, 2020, p. 10). Another said that he used Red Bull in the early hours of the morning (around 3 am), when he became unable to hear certain frequencies.

Kamps also talked with night workers about what they did to catch some sleep after work, especially after using amphetamines (the effects can last for around 8–12 hours, see Keogh 2010). His informants told him that, to ensure sleep, they stopped taking amphetamines around 2–3 am and smoked some cannabis once they got home. Others used melatonin to reset their biological clocks. But other factors also could keep them awake. If a performance or event went particularly well, the excitement could still be palpable after returning home. For the event organizers, whether money was made or lost greatly affected their state of being; returning home, they often sat on the couch, contemplating, with images of the evening going through their heads, what went well and what went wrong. These emotions, together with the rhythm of their internal body clocks and the chemical stimulants still coursing through their bodies, made it hard to fall asleep, even if they actually felt tired. Andy explained that one could be exhausted yet unable to fall asleep:

You might lay in bed at seven in the morning, be half asleep for three hours and then be fully awake again around eleven.” He would then get out of bed. Bart described the same phenomenon: “Your body is fully awake but your brain is lagging behind.” (Kamps and Hymans forthcoming, 2020, p. 11)

Our fieldwork in leisure sites in Amsterdam and Brooklyn showed that these nightlife workers were cautious users of stimulants, striving to meet the various demands of their nighttime work. Many used cocaine, which has as an advantage that its effect fades fast, allowing the workers to sleep when they get home. But amphetamines, in the form of ADHD drugs or speed, were also used often, and these made sleeping harder. In addition, several workers managed their stamina with energy drinks, which generally contain around 50 mg of caffeine per 250 ml (similar to a cup of coffee).

While moderating their stimulant use, and complementing it with other substances such as cannabis and melatonin to sleep, many of the interlocutors admitted to feeling burned out over time, which they attributed not only to their substance use but also simply to the lack of daylight in their lives.

## Long Working Hours in Physically Demanding Work

In the Philippines and Indonesia our focused ethnographies examined how young people used stimulants to have energy and strength enough to conduct physically challenging work, laboring long hours as porters, construction workers, and security guards. The use of amphetamines as fuel for labor in Asia has been observed by Sherman and colleagues (2008), who describe how methamphetamines—called *yaba* (crazy drugs) in its pill form in Thailand, and *shabu* in its powder form (also known as crystal meth) in Indonesia and the Philippines—entered the market in the 1990s to enhance performance in physically demanding roles. A young construction worker in Thailand reports:

I felt I could work more and earn more as well… When we delivered cement powder, we got 400 baht for one trailer… If we didn’t take *yaba* we would be able to deliver only one or two trailers. But when we took *yaba* we became diligent. (Sherman et al. 2008, p. 42)

Participating in the ChemicalYouth project, Lasco examined the use of *shabu* among unemployed young men hustling for work in a Philippines harbor city. They worked as *tambays* (stand-by) porters in the harbor, vendors to the bus passengers that frequent the port, and occasionally at night as sex workers. Their work comprised of long hours hanging around till boats and buses arrived. Some of them combined working as a porter with selling freshly roasted peanuts and drinks to bus passengers. Lasco learned that their work as street vendors also, ocassionally, involved stealing mobile phones from travelers, joining in “car-napping operations,” and engaging in sex work.

Lasco described how his informants killed time while “standing by” by smoking cigarettes, as many as 10–20 cigarettes a day. *Shabu*, they asserted, provided them with confidence and stamina, and reduced their inhibitions, allowing them to engage in a multitude of services. One told Lasco (2014), “We are not educated and we have nothing. Where will we get the confidence to talk to others, if not from *shabu*?” (p. 785). Young men were initiated into *shabu* inhaling by their *barkada*, which is both a social and economic group consisting of peers. The cost of one small sachet of *shabu*, which can be used by three people, is 500 pesos (US $12.5); the paraphernalia, consisting of three bits of aluminum foil and a lighter to heat the substance, was also shared. Everyone in a *barkada* uses *shabu*, which expresses loyalty, thereby forging social bonding in the group. “Scoring” sessions take place in ordinary port houses called “*pwesto*” (from the Spanish “*puesto*,” or place). The owner of the *pwesto* collects 20 pesos from the users, in exchange for providing them with the security of being in a private home where the substance can be used away from the gaze of the police. The young men said that, after an immediate euphoria, they experience a sustained effect of being “*kalmado*” (calm) and “*ganado*” (enthusiastic). They said that using *shabu* boosted their skills, abilities, and industriousness. Like Kamps’s informants in Amsterdam, they treated any insomnia caused by their *shabu* use with cannabis and Red Horse beer, which they routinely used to fall asleep. Several evenings a week, the *barkadas* gathered at night to drink alcohol together (*inuman*); this was leisure time. The costs for these evenings were shared by the whole *barkada*, unless someone was celebrating his birthday, in which case, the celebrant was expected to foot the bill. In the *inuman*, Red Horse often gave way to harder alcohol, such as a brandy called “Empi Light” (Emperador Light, 30% alcohol).

Alerted to the precariousness of his interlocutors’ lives, Lasco (2013) calculated how much they spent using multiple substances, as well as how much they earned from various sources. The results are presented in the Table 6.1. His findings reveal that the substances cost more than what was actually earned; the shortfall was met by occasionally doing sex work (earning around 500 pesos for oral sex services provided to gay men, and more if anal sex is done), or stealing and selling a mobile phone.

**Table 6.1:** Weekly drug expenses vs. weekly income of seven drug users (in Philippines pesos; 50 pesos equals US $1) (Lasco 2013, p. 81)

Weekly expenses	1	2	3	4	5	6	7
Shabu	1400	1400	1400	1000	1400	600	800
Cannabis	140	140	100	100	40	60	60
Cigarettes	262	262	175	175	175	175	150
Alcohol	280	280	200	280	280	280	200
Total	2082	2082	1875	1555	1455	1090	1210
Weekly income	1050	1050	1400	1400	1050	1050	1050

Lasco writes that, while most of his informants used *shabu*, there were also a few young men who resisted doing so. One of them explained that users often lost the ability to distinguish between using in order to work, and working in order to use (Lasco 2013). The users asserted that the greatest danger in using *shabu* was its illegality, speaking of weekly raids, youths getting jailed, and worries about their safety. They nevertheless perceived the benefits to outweigh the costs. In publishing this case study, Lasco decided to not publish the name of the city where he did fieldwork, and we followed his example in this book, worrying that Philippine drug authorities might crack down on the young men working in the harbor. Little did Lasco know that just a year later Rodrigo Duterte would be elected president, and that he would declare a war on *shabu*, giving local police officers the green light to shoot any drug user without due process.

Another focused ethnography in the Philippines was conducted by Leo Diego, who examined stimulant use among 21 security guards in Puerto Princesa, Palawan (Diego 2017). Providing security is the most common job for young men in the Philippines, and security guards are an omnipresent feature of contemporary Philippine society, seen everywhere, in schools, malls, banks, and other big establishments where people frequent. Diego observed that being alert during their long shifts was a key challenge for these workers, and that they relied on energy drinks as the main substance they use to help stay awake.

The majority of the participants entered the security industry because of their lack of college degrees; most of them were high school graduates. They worked at pawnshops, banks, department stores, hotels, and schools. Work circumstances differed at these sites. The pawnshops demanded a high level of security, as money was exchanged there and had to be protected. Security guards in pawnshops were bound to follow a no-sitting policy while on duty. Thus, alertness was crucial: as guards, they had to check the body language of customers, distribute forms to be filled out, and make sure that no trouble transpired. Banks also hired security guards for the same reason—money matters. Working in a bank was full of tension, especially for guards who drove armored vans. With their lives at stake, guards had to pay utmost attention to every movement in their surroundings. On the university campus, the work of security guards was very different. With its almost 20,000 students, Palawan State University was the largest tertiary educational institution in the province, and security guards were needed to check that students entering the premises had proper identification: no ID, no entry.

In general, the work of security guards is undervalued in the Philippines, with some seeing it as a job for lazy people. Many people believe that security guards just stand there, not thinking, all day at their posts. For example, Roland, a security guard who worked at a bank, always heard that being a security guard was a job for a bystander, because guards not only just stand and walk at their posts, but can also sleep if no one is watching. Roland explained that, because of this misperception, some people from his neighborhood said that when you applied for a security job, it must be your last choice. But Ric, a security guard at a mall, defended his work: guards must be conscious of everything that is happening in their vicinity at all times. They must constantly be reading the body language and facial expressions of the people around them. Their minds must always be aware of what is to be done in case of unexpected trouble. They must be observant at all times. The greatest challenge reported by security guards was maintaining their alertness and fighting off sleep. This challenge had to do with the long and irregular working hours assigned to security guards. Three guards often shared a single post; each of them had to have a shift of eight consecutive hours, and their work schedules could change with little or no notice.

Despite the stress involved, being a security guard was not a well-paid job. Most security guards in Puerto Princesa earned the minimum wage of US $5 per day, or approximately US $140 per month. Many paid rent to a boarding house, which in Puerto Princesa cost at least US $30–60 a month, excluding water and electricity. Most guards commuted to work (spending US $0.40 daily for fare) and bought their lunch (US $1). Most of our interlocutors also supported families and other dependents, and struggled in the face of the rising costs of basic necessities, such as rice, fish, and gasoline. Many fell into debt, and lived in a perpetual struggle to pay their bills and promissory notes. The precarious economic situation of most security guards meant that they depended on keeping their jobs, and any absence from work meant that they lost pay. Furthermore, many feared being caught sleeping on the job, which could result in an instant dismissal. Many security guards thus resorted to chemical stimulants, which they called “*pampa*-alert,” their term for something that can be used to foster alertness.

Generally, “to be alert” meant being aware of anything in the vicinity. Troubles and crimes could come along at any time. In this manner, the guards’ lives were at risk, as they were expected to protect people and the establishment for which they are responsible. Most security guards were men, and most were on duty at night. Some security agencies told us that female guards were not permitted to work the night shift, because, they said, female guards were not on par with male guards in terms of alertness and self-defense. But still, female guards were often found at the entrances of department stores and malls, to name a few.

Fourteen of the security guards interviewed by Diego drank instant coffee to keep themselves awake (Nescafe 3-in-1, Great Taste, and Kopiko Brown are popular; all contain large amounts of sugar), and 12 of them also drank energy drinks during their shifts, see Table 6.2.

**Table 6.2:** Kinds of stimulants used by security guards in Puerto Princesa (*N *= 16)

Name	Post	Sex	Energy Drink	Cigarette	Coffee
Joker	Bank	Male	Cobra energy drink during night duty	Fortune White cigarettes, 1 pack per week	Nescafe 3-in-1 coffee
Jerry	Bank	Male	Cobra on duty	Nonsmoker	Great Taste coffee (no side effects)
Mantal	Bank	Male	Cobra almost every day	Nonsmoker	Great Taste (no side effects)
Roland	Bank	Male	Not using energy drinks	2 packs per day	Nescafe 3-in-1
Ric	Department store	Male	Cobra, Sting, Red Bull, Extra Joss every week	2 packs of Fortune every other day	Kopiko Brown coffee every day
Bryan	Department store	Male	Cobra during the day	Nonsmoker	Nescafe 3-in-1, thrice a day
Netz	Department store	Female	Cobra during the day	2 cigarettes per day	Great Taste twice a day
Ged	Hotel	Male	Not using energy drinks	Nonsmoker	Kopiko Brown twice a day
Charm	University	Female	Cobra during night duty	Nonsmoker	Creamy latte- flavored coffee twice a day
Ems	University	Female	Cobra during day shift	Nonsmoker	San Miguel coffee twice a day
Salad	University	Female	Cobra during night duty	2 cigarettes per day	Great Taste twice a day
Ann	University	Female	Cobra during the day	Nonsmoker	Great Taste every morning
Jacky	Mall	Female	Not using energy drinks	Nonsmoker	Kopiko Brown every morning
Vicky	Mall	Female	Not using energy drinks	Half-pack a day	Great Taste every morning before duty
Allan	Pawnshop	Male	Cobra every other night	Winston, 3 cigarettes a day	Great Taste
Mag’z	Pawnshop	Male	Cobra every night duty	Nonsmoker	Not drinking coffee

Table 6.2 shows the popularity of the Cobra energy drink, which was used by 12 of the 16 informants. Five of the Cobra drinkers specified that they used the energy drink during night shifts. It also shows that half the informants did not smoke cigarettes; the reason for this is the strict anti-smoking policy in Puerto Princesa, where smoking is only allowed in designated areas.

**Fig. 6.1:**
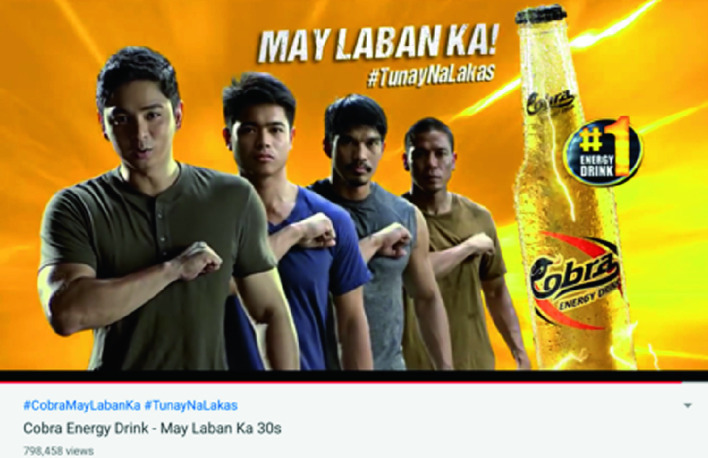
Screenshot of Cobra advertisement (*Source* Photo taken by Anita Hardon, 2019, the Netherlands)

A 350 ml bottle of Cobra costs US $0.40 and contains 134 mg caffeine (the same amount as two cups of coffee; this is about double the content of the energy drinks sold in Amsterdam). It also contains B vitamins, ginseng, and sugar. Cobra is the market leader in the energy drinks segment of the beverages market in the Philippines, accounting for 74% of sales (Euromonitor International 2020). The brand’s bottles are sold in *sari*-*sari* stores (informal neighborhood shops) and the brand is heavily advertised on TV and through Twitter, Facebook, and YouTube. Everyone in the Philippines knows Cobra’s slogans: “*May Laban Ka*” (you’re up against it) and “*Tunay na Lakas*” (full of strength). Its popularity is attributed to the fact that it is promoted by the award-winning Filipino actor Coco Martin (the attractive muscular guy on the left in below advertisement) (Fig. 6.1).

**Fig. 6.2:**
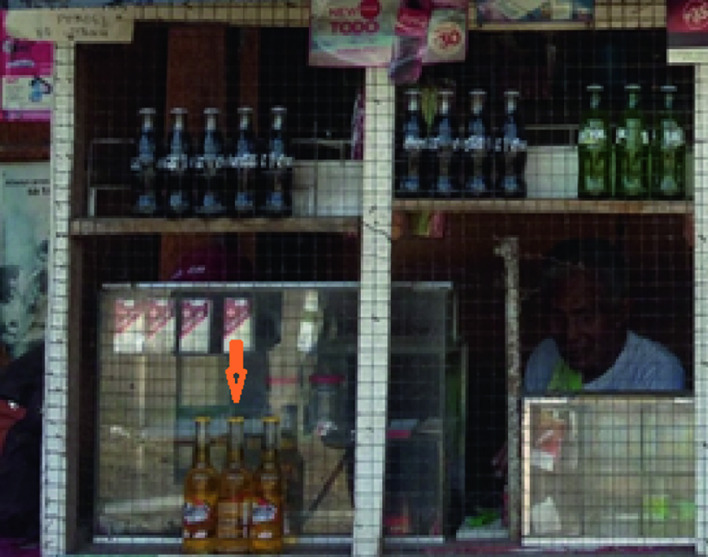
*Sari*-*sari* store selling bottles of Cobra (yellow liquid toward the front of the window) (*Source* Photo taken by Anita Hardon, March 2014, the Philippines)

See Fig. 6.2, for an image of a typical neighborhood outlet for Cobra; notice how Cobra is placed prominently in the store along with Coca Cola, Sprite, and Fortune cigarettes.

Despite the popularity of this energy drink, users’ experiences are mixed. Bryan, a 25-year-old security guard at a department store, told us that sometimes when he uses Cobra he felt wide awake, but there were also times when he couldn’t help falling asleep. Charm, a female guard at a university, said that Cobra caused her to suffer a urinary tract infection. When she stopped using Cobra, she just walked around her post in order to avoid feeling sleepy while on duty.

Only a few of the security guards admitted to using *shabu* to stay alert during the night shift, perhaps because Puerto Princesa has a zero tolerance policy for drug use. One of the guards, Joker, told us of how he used *shabu* when he needed to serve as a shift reliever, meaning he had to be at his post almost 22 hours in a row. He also shared that there was a popular drug called Fly High that could also be used to stay alert and was also effective in increasing stamina during sexual intercourse. He said that this drug was a combination of methamphetamine, Viagra, and ecstasy, and could be bought for around US $30–60 per tablet. When asked about the effect of the drug, Joker responded, “For three to four days you will not feel hungry, your being awake is continuous, your awareness will be active, you are unstoppable, and you have more stamina for sex” (Diego 2017, p. 75).

**Fig. 6.3:**
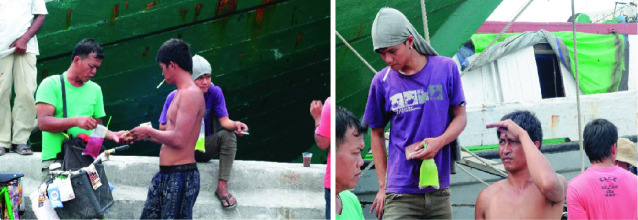
Dock workers buying an energy drink from a local vendor—break time (*Source* Photos taken by Sari Damar Ratri, October 2015, Indonesia)

For our final case study, we turn to the use of stimulants by two categories of low-income workers: porters and construction workers in Makassar, Indonesia. This research was carried out by Amalia Anwar (2017) in infrastructure development locations (such as housing and offices) that employed construction workers and in the seaport with dockworkers. The manual laborers involved in this study were 30 construction workers (15 men and 15 women) and 20 dockworkers (all men), aged between 17 and 24 years (Fig. 6.3).

**Fig. 6.4:**
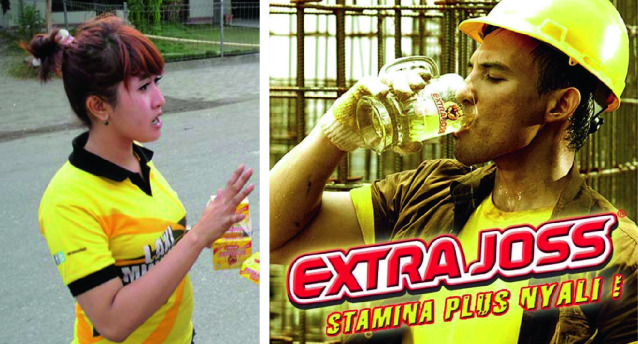
Left: sales promotion girl selling a box of six sachets of Extra Joss for 5000 Rupiah (US $0.36) in the port of Makassar; across her breasts is printed the slogan *Laki minum* (male drink) (*Source* Photo taken by Anita Hardon, May 2014, Indonesia; right, screenshot of an advertisement for Extra Joss from the website Shopee with the slogan “Stamina plus *nyali*” [stamina plus courage] Shopee [2020]. https://shopee.com.my/Extra-joss-energy-drink-10-boxes-per-package-i.110261307.1937551005)

Energy drinks, which are classified as “health drinks” in Indonesia, were the most commonly used stimulants in these fieldwork sites. Kuku Bima and Extra Joss are popular local brands, sold in sachets (US $0.06/sachet) in groceries and informal stalls, and mixed with water by vendors (as illustrated in Fig. 6.2). Advertisements for such drinks in various print and electronic media are plentiful and varied, with provocative slogans such as “*lelaki pemberani*” (brave man), “*jiwa laki bukan pengecut*” (men’s souls not cowardly), and “ Extra Joss, perfect goal” (aired during the world football championships) that stimulate men to buy and consume them. The sachets are also marketed by sales promotion girls, see Fig. 6.4.

The ad for KukuBima Ener-G drink features Ade Rai, an athlete and artist, and shows a picture of his muscular body, evoking strength and vitality, accompanied by a picture of ginseng, which is known for its virility enhancement effects.

The slogans of such ads promote not only masculinity—for example, with slogans like these: (This is the King, Brave Man, Energy of the Brave, Stamina Plus Courage, For Men Trusted Men, Men’s Souls are not Cowards); and strength (Restores Tired Stamina, Stamina for the Country, Fresh, Full of Energy, Real Energy)—but also competition (The Essence of Football, Secret of the Champions, Premier Energy Drink in the World).

Although advertising plays an important role in promoting the use of energy drinks, workers generally learned about energy drinks from their coworkers who first consumed them. Table 6.3 lists the contents of the energy drinks that were popular among manual laborers we spoke with. Note how all three of the popular brands contain 50 mg of caffeine per sachet along with ginseng, different kinds of vitamin B, flavors, and sweeteners (honey and/or sugar). The main difference is taste.

**Table 6.3:** Nutritional information on packages of popular energy drinks

No.	Name	Manufacturer	Contents	Benefits
1.	KukuBima Ener-G	PT. Sido Muncul	300 mg ginseng powder, royal jelly 30 mg, honey 100 mg, taurine 1000 mg, caffeine (1,3,7 trimethylxanthine) 50 mg, vit. B3 20 mg, vit. B6 5 mg, vit. B12 5 mcg, flavor, Brilliant Blue CL 42090	Restores stamina, body metabolism and refreshes body
2.	Extra Joss Active	PT. Bintang Toedjoe	Taurine 1000 mg, 350 mg ginseng powder, vit. B2 3 mg, vit. B3 16 mg, vit. B5 5 mg, vit. B6 1.5 mg, vit. B8, 10 mg, vit. B9 100 mcg, vit. B12 1 mcg, royal jelly 2 mg, 1,3,7 trimethylxanthine (caffeine) 50 mg	Helps maintain stamina and refreshes body
3.	Kratingdaeng	PT. Asia Health Energy Beverages under license from TC. Pharmaceutical Industries, Co., Ltd.	Taurine 1000 mg, caffeine 50 mg, inositol 50 mg, niacinamide 20 mg, pyridoxine HCL (vit. B6) 5 mg, dexpanthenol (Pro-vitamin B5) 5 mg, cyanocobalamine (vit. B12) 5 mcg, sugar (25 grams of pure sugar), Ponceau flavoring	Supplement drink. Helps refresh body during hard work or exercise

Limited rest periods and the heavy workload made it difficult for manual laborers to take a lunch break, so consuming energy drinks became a substitute for lunch. Surti (24-year-old construction worker), for example, felt full and energetic after consuming KukuBima Ener-G, even when she had not eaten. Ulla, a 24-year-old dockworker, admitted to frequently consuming KukuBima Ener-G (grape flavor) while resting in order to restore her stamina before her next shift. Ella and her coworkers would regularly consume such energy drinks in large volumes, often mixing five or six sachets with a jug of cold water.

Energy drinks were used not only to work during the day but also for overtime shifts. Foremen regularly asked workers to work 12 hours a day and to continue working over the weekend to get the job done. Ariana, a 23-year-old construction worker, said she drank energy drinks (Extra Joss Active) only when working overtime, after a 12-hour shift. For some of our interlocutors, energy drinks were panaceas, used to overcome body aches, increase appetite, and improve sleep quality.

The construction workers preferred KukuBima Ener-G. Their foreman bought the sachets for them, and deducted these costs from their salary. Ratih, a 20-year-old, explained:

In the workplace, they buy it for us, usually bought in the morning, and also the afternoon, the foreman notes it. Usually we make one box of grape flavored KukuBima Ener-G mixed with milk, mixed with water in a jug, and we drink it together. (Anwar 2017, p. 196)

Energy drinks were consumed not only for work but also for fun. After receiving their wages, the construction and dock workers would gather and hang out with friends, staying up late watching movies together while eating junk foods and drinking energy drinks. Energy drinks were mixed with milk or with cheap alcohol. Our interlocutors explained that the alcoholic mixes made them sleep well, relieved body aches, and strengthened them for work the next day.

The widespread use of energy drinks for both work and recreation in our field sites in Makassar meant that the workers had a relatively high intake of caffeine. We wondered: did our interlocutors care about the dangers of consuming energy drinks? Sari, a 24-year-old construction worker, claimed she did not know about the high level of caffeine, and was surprised that energy drinks were considered harmful in excess. Ruslan, a 24-year-old dockworker, said that he had tried to consume a M-150, but experienced heart palpitations. Although Extra Joss Active did not have a negative effect on his body, he preferred KukuBima Ener-G because of its grape flavor, which he found delicious. Asril, a  20-year-old construction worker, moved from one energy drink brand to another. While he found Extra Joss Active tasty, he, like Ruslan, preferred KukuBima Ener-G because of its grape taste; for him, Kratingdaeng was not an option because it caused a variety of negative effects: heartburn, dizziness, stomach ulcers, and nausea. Kamaruddin, a  20-year-old dockworker, also chose KukuBima Ener-G rather than Extra Joss Active, because Extra Joss Active caused a burning sensation in his throat every time he consumed it.

Among those concerned about the side effects of regularly consuming energy drinks, some had reduced their energy drink consumption, and some were looking for alternatives to overcome tiredness. Jamal, a 20-year-old construction worker, had limited his consumption of energy drinks, replacing them with other cold drinks that contain lots of sugar (like Teh Gelas) or water. Others quit consuming energy drinks cold turkey. Muhlis, a 24-year-old dockworker, for example, stopped consuming all energy drinks because he often experienced stomach pain and heat in his throat. He tried to prevent these adverse effects by consuming plain soda water mixed with milk and Pilkita—an analgesic drug sold as a “strength drug”—because he believed that the mixture could relieve his pain. Ulla, a 24-year-old dockworker, had replaced energy drinks with a mixture of soda water and raw chicken eggs.

## In Conclusion

Scholars of youth have described how young people use stimulants—mostly ADHD drugs—to work long hours to keep up with academic requirements, a practice that is considered problematic because it is an “ off-label” use and because it may create academic advantage in allowing users to unfairly enhance their performance (Coveney et al. 2011; DeSantis and Hane 2010; Garnier et al. 2010; McCabe et al. 2006). However, young people’s use of other stimulants like energy drinks to increase productivity has received much less scholarly attention. This is remarkable given the high sales of energy drinks across the globe, as well as the health risks associated with their frequent consumption.

One of the rare studies that examines the use of energy drinks by youth found that nearly one in five students of a secondary school in Ontario consumed energy drinks to be alert (Reid et al. 2015). Another web-based study conducted across campuses in the United States found that almost half of the 667 respondents had used energy drinks. This study reported that energy drink use was associated with poor sleep and tiredness the next day (Patrick et al. 2018), an observation also made in a study of energy drinks by US soldiers in Afghanistan (CDC 2010). Researchers at the Center for Food Policy and Obesity at Yale University (Pomeranz et al. 2013) view the increasing use of energy drinks by youth in the United States as a public health hazard, because the high levels of caffeine in the drinks can lead to caffeine overdose. In addition, they warn that these products contain both high levels of sodium and novel ingredients, such as taurine, guarana, and ginseng, the combined effects of which have been understudied. Finally, they point to the heavy advertising of these products to youth through digital media, Facebook, and sports events and brands.

Our approach of examining situated chemical practices allowed our youth ethnographers to observe both the use of stimulants and the use of chemicals to address sleep problems. Across our sites in Amsterdam, Brooklyn, Puerto Princesa, and Makassar, we found that patterns of stimulant use differed. The porters in the Philippines earned money not only by carrying wares from buses to boats and back. They also earned money by selling goods and sex. For this interactional labor, they valued the confidence enhancement that amphetamines offer. The security guards whom we interviewed in Puerto Princesa sought products to keep them awake during their night shifts. For them, energy drinks did the trick. But they earned very little money, and energy drinks took a substantial part of their limited income, which forced them to moderate their use. In Indonesia, energy drinks are very cheap, and people used them as a panacea, not only to have stamina at work but also for virility and for bodily aches, and they valued the flavor that energy drinks brought to mixed alcoholic drinks. But the consumption of the caffeine in these drinks can lead to adverse effects, such as heart palpitations and nausea. Several of our interlocutors were trying to quit using energy drinks for these reasons. In Amsterdam, our ethnographic research found that nightlife workers also used amphetamines to stay alert, but they could easily get ahold of ADHD drugs for this purpose. Moreover, in both Brooklyn and Amsterdam night workers often used cocaine, which is much more expensive than crystal meth, to enhance their work performance. Both ADHD drugs and cocaine have less potential for addiction and less severe adverse effects than crystal meth (NIDA 2019). In both cities, users moderated their intake to manage sleep patterns, though the tiredness reported among students in the United States and soldiers in Afghanistan is a common feature of the ChemicalYouth narratives of our interlocutors.

Harm reduction programs, designed to mitigate drug harms, fail to recognize the health risks, including tiredness and sleep problems, related to the widespread use of energy drinks and other legal stimulants by youth (Hardon and Hymans 2016). Instead, such programs focus on risks of recreational drugs such as heroin, cannabis, and cigarettes and their potential for addiction (see also Chapter 10.1007/978-3-030-57081-1_9). However, since stimulant use for work and school is arguably a bigger problem than recreational use, as it takes place every working/school day and not only occasionally during free time, it is surprising that this area has been overlooked. This everydayness of use adds to the health risks.

Our field studies suggest that, to address these health risks, student and occupational health programs are needed to not only inform youth of the risks of using stimulants frequently but also to address the study demands and work conditions that give rise to the need for these drugs in the first place. Academic cultures place high demands on students, normalizing night work. Participating in stress-inducing academic cultures, working the night shift as security guards and club staff, and putting in long hours doing heavy labor on construction sites and in harbors—all of these fuel demands for energy drinks and amphetamines to stay alert, to have the stamina, and to feel the physical strength needed for the job.

The tiredness and sleep problems experienced by our interlocutors need to be remedied by better work conditions, as pointed out by Costa (2010) and Wolf-Meyer (2011), who recommend that shift schedules should be designed to reduce stress and adverse effects on health, and to minimize circadian disruption, sleep deficits, and fatigue. Sufficient time for recuperation after night shifts is needed, as is decent compensation for heavy and irregular work. Workers engaged in hard, physical labor could be offered time to rest and provided with nutritious meals, rather than energy drinks, to fuel their bodies and give them time to recover. Our study of young workers’ situated chemical practices suggest that the widespread use of stimulants threatens to disrupt their circadian regimes even further, causing potentially severe metabolic disorders, especially when they use such products on a daily basis because they cannot afford to take a rest.

## ChemicalYouth Ethnographers

**Fig. 6.5:**
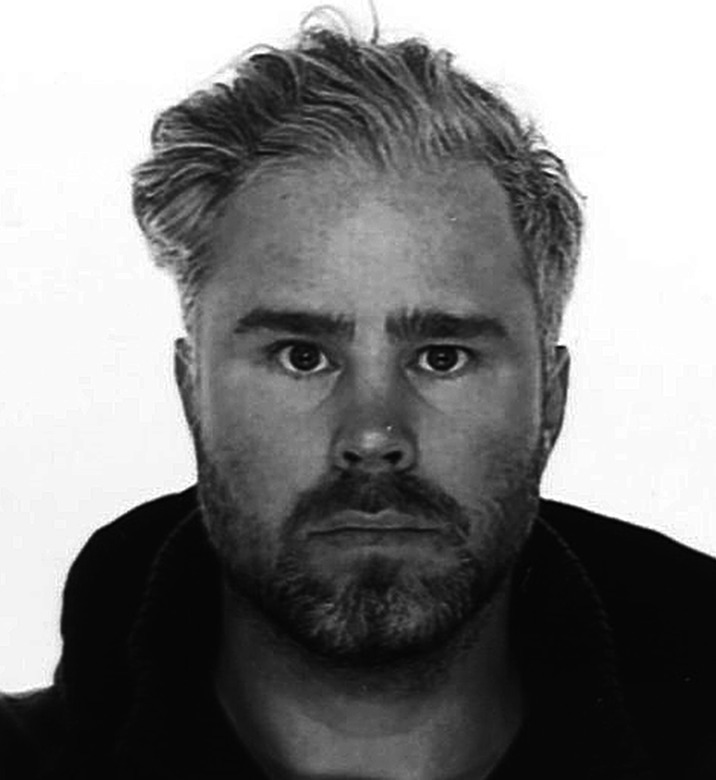
Daan Kamps

Daan Kamps graduated from the Research Master’s program in Social Sciences at the University of Amsterdam. His current research interests lie in urban nightlife work, chronobiology, and drug use, which he studied as a researcher for the ChemicalYouth project. For more than five years, he has been active within the Amsterdam club circuit as a DJ and event organizer, which provided him the opportunity to become familiar with the field (Fig. 6.5).

**Fig. 6.6:**
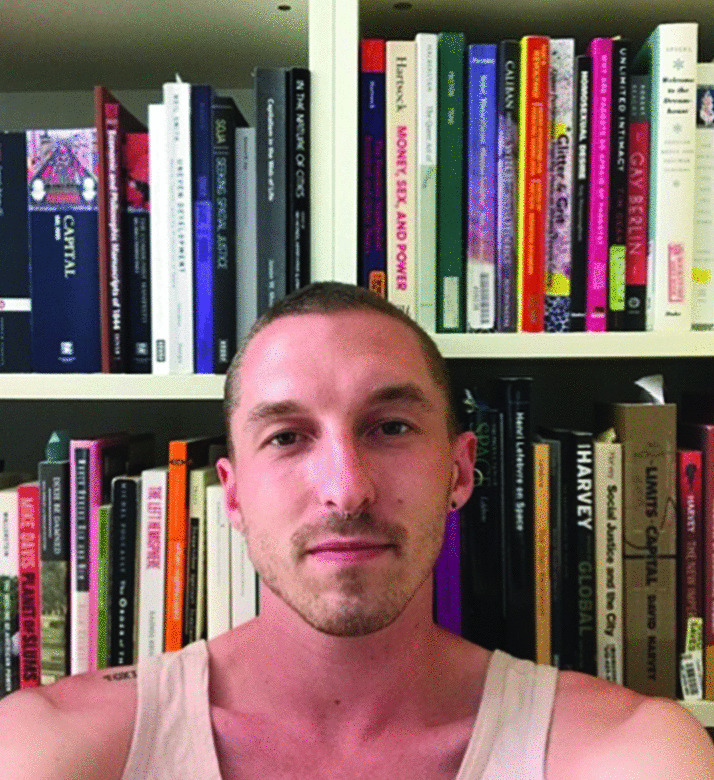
Tait Mandler

Tait Mandler carried out research in Brooklyn, New York, on the lives of workers in queer nightlife spaces. Their research explored the ways that workers use chemicals to get their jobs done and how they care for themselves and others (Fig. 6.6).

**Fig. 6.7:**
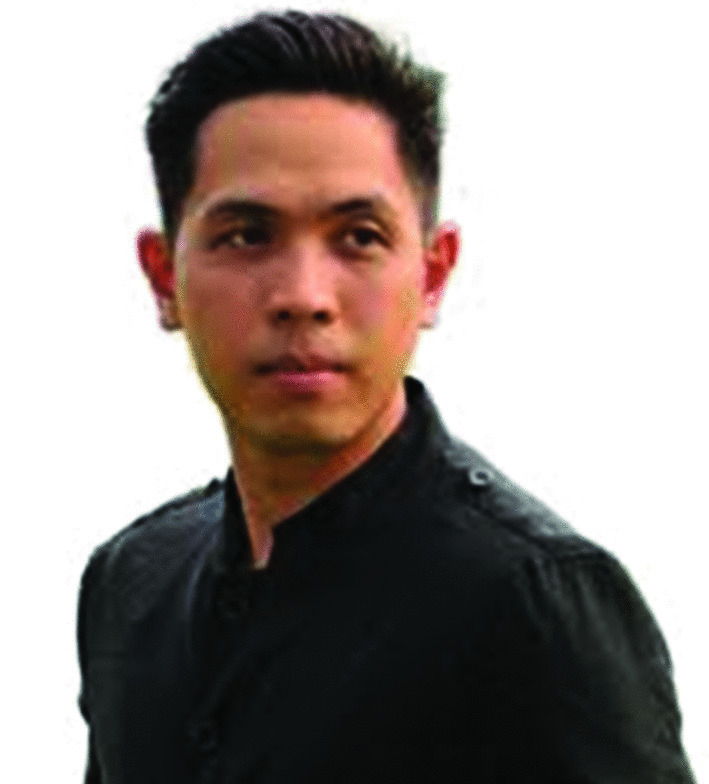
Gideon Lasco

Gideon Lasco is a physician and medical anthropologist. He obtained his Ph.D. from the University of Amsterdam and his M.D. from the University of the Philippines, where he currently teaches anthropology. His research examines the chemical practices of young people, the meanings of human height, the politics of health care, and the lived realities of the Philippine “ drug war.” A Palanca-winning essayist, he maintains a weekly column in the *Philippine Daily Inquirer*, where he writes about health, culture, and society (Fig. 6.7).

Leo Diego was a researcher for the ChemicalYouth project at the Palawan Studies Center at Palawan State University and conducted fieldwork on security guards’ chemical practices in the Philippines.

Ahsani Amalia Anwar was a researcher for the ChemicalYouth project at the Palawan Studies Center at Palawan State University and conducted fieldwork on the social lives of energy drinks among physical laborers in Indonesia.
